# Can weekend warriors and other leisure-time physical activity patterns reduce the atherogenic index of plasma (AIP)? A cross-sectional analysis based on NHANES 2007-2018

**DOI:** 10.3389/fendo.2025.1511888

**Published:** 2025-03-06

**Authors:** Yueyue Niu, Xingjuan Chen, Ling Feng

**Affiliations:** Cadres Health Protection Department, Guang’anmen Hospital, China Academy of Chinese Medical Sciences, Beijing, China

**Keywords:** physical activity, weekend warriors, regular activity, atherogenic index of plasma, NHANES

## Abstract

**Background:**

With the shift in modern lifestyles, the relationship between physical activity (PA) and health has emerged as a significant concern in global public health. A sedentary lifestyle poses a substantial threat to cardiovascular health, particularly through the development of atherosclerosis, the primary pathological basis of cardiovascular disease (CVD) and a condition influenced by various lifestyle factors. The atherogenic index of plasma (AIP), a critical indicator for predicting cardiovascular disease risk, assesses an individual’s risk of atherosclerosis by reflecting the ratio of high-density lipoprotein cholesterol (HDL-C) to triglycerides (TG). Despite the recognized importance of PA, the impact of various physical activity patterns on AIP remains unclear.

**Methods:**

This study utilized the National Health and Nutrition Examination Survey (NHANES) database from the United States. PA was assessed via a questionnaire, and participants were categorized into four groups: inactive, insufficiently active, weekend warriors (WW), and regularly active (RA). The AIP was calculated via the ratio of HDL-C to TG, with covariates such as age, sex, race, and body mass index controlled. Multivariate regression analysis served as the primary analytical method.

**Results:**

This study included a total of 24,504 participants. After adjusting for all potential covariates, RA (β=-0.044, *P*<0.0001) was associated with a significant reduction in AIP compared with WWs (β=0.01, *P*=0.65). Additionally, subgroup analysis and interaction tests showed that the PA-AIP association varied slightly among individuals with different education levels (*P* for interaction = 0.07) and marital statuses (*P* for interaction = 0.09), although these differences were small and did not reach statistical significance. Furthermore, restricted cubic splines (RCS) analysis revealed a significant, nonlinear, and negative correlation between total weekly PA and AIP among inactive individuals (*P*<0.001, nonlinearity *P*<0.001). The study found that 510 minutes of total physical activity per week is a threshold, beyond which the rate of decrease in AIP tends to slow down.

**Conclusion:**

RA is more effective in reducing AIP than WWs are. For inactive adults, engaging in more than 510 minutes of PA per week significantly reduces the AIP.

## 1 Introduction

With the rapid acceleration of modern life and the continuous rise in stress, the close relationship between physical activity (PA) and health has emerged as a central issue in global public health. In this context, sedentary lifestyles are becoming increasingly prevalent in modern society, posing a significant threat to cardiovascular health (1, 2). Atherosclerosis, the pathological foundation of cardiovascular disease (CVD), is profoundly influenced by various lifestyle factors (3–5), underscoring the urgent need to explore the relationship between physical activity patterns and atherosclerosis risk.

The atherogenic index of plasma (AIP), a crucial biomarker for assessing cardiovascular disease risk, serves as a scientific basis for predicting an individual’s potential risk of atherosclerosis by precisely reflecting the ratio between high-density lipoprotein cholesterol (HDL-C) and triglycerides (TG) (6). A high AIP value is often indicative of increased cardiovascular disease risk, a finding validated by multiple studies (7–9). However, despite the well-recognized importance of physical activity, research on how different physical activity patterns specifically influence AIP remains limited, particularly in the context of large-scale, nationally representative samples. To address this gap, it is hypothesized that different physical activity patterns are significantly associated with AIP, and that certain patterns may have a more pronounced effect on reducing AIP.

In light of this, the present study leverages the comprehensive and authoritative National Health and Nutrition Examination Survey (NHANES) database in the United States, with the aim of systematically analyzing and elucidating the complex associations between different physical activity patterns and AIP. As a long-term, continuously updated national health monitoring program, the NHANES provides a dataset with broad and deep coverage across multiple dimensions, including lifestyle, physical activity, dietary habits, and mental health. This comprehensive dataset offers invaluable resources for exploring the potential benefits of physical activity on cardiovascular health. The study seeks to elucidate the impact of different physical activity patterns on AIP, thereby providing strong scientific support for the development of more precise and effective public health strategies and for the prevention of cardiovascular disease onset and progression.

## 2 Materials and methods

### 2.1 Study design and population

The NHANES is a key research initiative focused on assessing the health and nutritional status of adults and children across the United States. It involves the extensive collection and detailed analysis of health data from nationally representative samples, offering valuable insights into the complexities and interconnections of various health parameters. Notably, the NHANES survey process strictly adheres to the approval of the National Center for Health Statistics (NCHS) Institutional Review Board, ensuring the ethical compliance of the research, with written informed consent from all participants being an essential prerequisite for conducting the study (10). Given the broad public accessibility and stringent quality control of the NHANES database, no additional ethical approval is required for analyses conducted via this database. This study focuses on detailed data from six consecutive NHANES cycles between 2007 and 2018, covering a total of 59,842 individuals, thus ensuring the breadth and representativeness of the sample.

**Figure 1:**
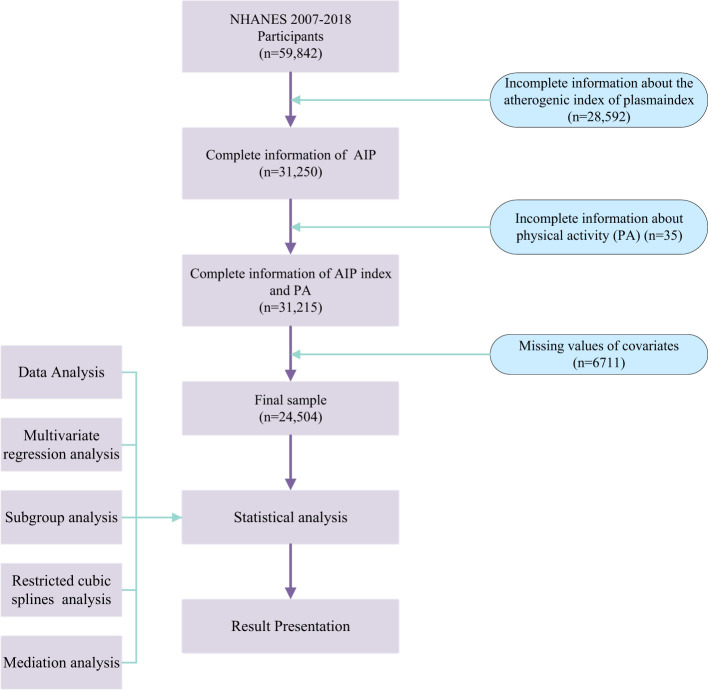
Study process overview. *Abbreviations*: NHANES, National Health and Nutrition Examination Survey; AIP, atherogenic index of plasma; PA, physical activity.

During the participant selection process, strict adherence to established criteria was maintained by excluding records with missing data on the AIP and PA, as well as incomplete information on other key covariates, to ensure the accuracy and reliability of the research results. Following rigorous screening, a total of 24,504 eligible participants were included in this study (**Figure 1**).

### 2.2 Physical activity

To assess PA levels, we used a PA questionnaire as the primary tool. This questionnaire inquires in detail about participants’ engagement in vigorous-intensity and moderate-intensity exercises, as well as physical exercise and leisure activities, each lasting at least 10 minutes continuously within a standard week (11). The weekly frequency of these activities (i.e., the number of sessions) was combined with the duration of each activity to precisely calculate the total PA for each participant. Additionally, based on the conversion factor from previous studies, 1 minute of vigorous physical activity (VPA) is equivalent to 2 minutes of moderate physical activity (MPA) in terms of energy expenditure (12). The formula total PA = 2*VPA minutes + MPA minutes was applied to assess total PA comprehensively. Based on participants’ self-reported leisure-time PA data, their activity patterns were subsequently classified into four categories (13, 14): first, the inactive group, consisting of participants who reported no engagement in either vigorous or moderate PA; second, the insufficiently active group, whose total weekly PA did not meet the recommended 150-minute standard; third, the weekend warrior(WW) group, which accumulated at least 150 minutes of total PA within a week, but this was mostly achieved in 1–2 sessions; and finally, the regularly active(RA) group, where participants completed at least 150 minutes of total PA across more than 2 sessions per week.

### 2.3 Atherogenic index of plasma

The AIP is calculated on the basis of the levels of HDL-C and TG in the blood, with the following mathematical formula: log10 [TG(mmol/L)/HDL-C(mmol/L)] (15). The AIP reflects an individual’s potential risk for atherosclerosis, and a higher AIP is closely associated with the development and progression of cardiovascular diseases (16).

### 2.4 Covariates

The selection of covariates was based on a comprehensive consideration of clinical expertise and prior research (17–19). This study included a range of covariates, specifically: age; sex (male and female); race (Mexican American, other Hispanic, non-Hispanic White, non-Hispanic Black, and other races); marital status (married or living with a partner, unmarried, divorced/separated/widowed); education level (less than high school, high school graduate or equivalent, college or higher); poverty income ratio (PIR) (low income: PIR ≤ 1.3, middle income: 1.3<PIR<3.5, high income: PIR≥3.5) (20); body mass index (BMI) (standard formula BMI = weight[kg]/height²[m²], categorized as<18.5, 18.5–25, 25–30,>30) (21); body roundness index (BRI) = 364.2 - 365.5 × √[1 - (WC²/(4π²))/(0.5 × height)²], where WC represents waist circumference (cm) (22); smoking status (never smoked, former smoker, current smoker); alcohol consumption (nondrinker, former drinker, heavy drinker, moderate drinker, light drinker) (23, 24); diabetes (based on diagnosis, fasting blood glucose≥7.00 mmol/L, HbA1c>6.5%, random blood glucose≥11.10 mmol/L, OGTT 2h>11.10 mmol/L, use of antidiabetic medications/insulin); and hypertension (systolic blood pressure≥140 mmHg or diastolic blood pressure≥90 mmHg, use of antihypertensive medications, physician diagnosis). The evaluation of cardiovascular disease (CVD) outcomes encompassed self-reported instances of congestive heart failure (CHF), coronary heart disease (CHD), angina pectoris, cardiac arrest, and cerebrovascular accidents (strokes). Individuals who positively responded to the inquiry about a physician’s prior diagnosis of CHD, CHF, angina, myocardial infarction, or stroke were subsequently categorized as having the respective CVD condition.

### 2.5 Statistical analysis

All the statistical analyses in this study were conducted via R software (version 4.2.3). The baseline characteristics of all participants are presented according to different PA pattern groups. Multivariate linear regression analyses were utilized to delve into the intricate relationships between PA patterns and the AIP, aiming to identify potential associations between these variables. Subgroup analyses were performed to explore variations in the impact of PA on AIP across different subpopulations. Interaction tests were utilized to assess whether potential confounding factors significantly influenced the PA-AIP association. Finally, restricted cubic splines (RCS) were applied to investigate possible nonlinear relationships between PA and AIP. A two-sided *P* value<0.05 was considered statistically significant.

## 3 Results

### 3.1 Essential attributes of the study participants at baseline

**Table 1** showcases a comparative analysis of the fundamental participant attributes across distinct PA pattern cohorts at the study. In-depth analysis revealed significant differences in sociodemographic characteristics, lifestyle factors, and health status indicators among the PA pattern groups. Specifically, participants in the WW group were predominantly male, with an average age of approximately 42 years, and were mostly non-Hispanic White. The majority of the study participants were either in a marital relationship or cohabiting with a partner, and they exhibited a higher educational attainment and possessed a greater PIR. With respect to lifestyle factors, WWs reported low alcohol consumption and a high prevalence of nonsmokers. Despite their engagement in physical activity, challenges in weight management were observed, as indicated by a higher BMI, placing them in the overweight category. However, in terms of BRI, the WW group, although higher than the RA group, had a lower BRI than the Inactive and Insufficiently Active groups. In terms of health status, participants in the WW group did not report chronic conditions such as diabetes, hypertension, or cardiovascular diseases.

**Table 1:** Participant demographics and initial data overview.

Variable	Total	Inactive	Insufficiently Active	RA	WW	*P* value
Age (year)	47.41 (0.26)	50.47 (0.29)	47.62 (0.36)	44.00 (0.36)	42.26 (0.62)	< 0.0001
Sex (%)						< 0.0001
Female	50.71 (0.01)	53.03 (0.66)	55.62 (1.09)	48.65 (0.62)	26.64 (1.78)	
Male	49.29 (0.01)	46.97 (0.66)	44.38 (1.09)	51.35 (0.62)	73.36 (1.78)	
Race (%)						< 0.0001
Mexican American	8.11 (0.01)	9.86 (0.98)	6.14 (0.62)	6.74 (0.65)	8.25 (1.06)	
Non-Hispanic Black	10.14 (0.01)	11.54 (0.90)	8.87 (0.67)	8.98 (0.65)	9.50 (0.90)	
Non-Hispanic White	69.11 (0.03)	65.51 (1.79)	73.18 (1.33)	71.81 (1.30)	69.56 (2.06)	
Oher	7.28 (0.00)	7.01 (0.45)	7.35 (0.58)	7.68 (0.50)	6.72 (0.78)	
Other Hispanic	5.37 (0.00)	6.08 (0.64)	4.46 (0.48)	4.79 (0.41)	5.98 (0.74)	
Marital status (%)						< 0.0001
Divorced, separated, or widowed	18.24 (0.01)	21.86 (0.53)	18.26 (0.80)	13.99 (0.54)	14.53 (1.65)	
Married or living with partner	63.67 (0.02)	63.10 (0.82)	65.71 (1.09)	63.75 (1.06)	61.40 (1.86)	
Never married	18.09 (0.01)	15.04 (0.59)	16.03 (0.82)	22.26 (1.01)	24.07 (1.62)	
Education (%)						< 0.0001
Completed high school	22.76 (0.01)	27.73 (0.73)	20.86 (1.03)	16.85 (0.71)	24.72 (1.73)	
Less than high school	14.55 (0.01)	22.44 (0.80)	9.67 (0.73)	6.99 (0.41)	11.19 (1.12)	
More than high school	62.70 (0.02)	49.83 (0.91)	69.48 (1.32)	76.16 (0.94)	64.09 (1.97)	
PIR (%)						< 0.0001
High income	43.82 (0.02)	33.33 (1.04)	49.20 (1.49)	54.74 (1.28)	45.81 (2.38)	
Low income	21.06 (0.01)	27.79 (0.88)	15.98 (0.83)	15.00 (0.68)	18.64 (1.50)	
Middle income	35.12 (0.01)	38.89 (0.70)	34.81 (1.19)	30.26 (1.01)	35.54 (2.05)	
BMI (%)						< 0.0001
Normal	27.75 (0.01)	22.60 (0.51)	27.46 (1.07)	34.32 (0.92)	30.27 (2.21)	
(18.5 to <25)						
Obese	37.94 (0.01)	45.00 (0.67)	39.21 (1.04)	28.78 (0.90)	32.79 (2.22)	
(30 or greater)						
Overweight	32.87 (0.01)	30.87 (0.64)	31.91 (1.09)	35.54 (0.79)	36.04 (1.92)	
(25 to <30)						
Underweight	1.43 (0.00)	1.53 (0.15)	1.43 (0.25)	1.37 (0.18)	0.90 (0.24)	
(<18.5)						
BRI	5.39 (0.03)	5.92 (0.04)	5.43 (0.05)	4.74 (0.04)	4.92 (0.09)	< 0.0001
Drinking (%)						< 0.0001
Former	12.64 (0.01)	16.65 (0.54)	11.06 (0.77)	8.63 (0.49)	9.08 (0.91)	
Heavy	21.69 (0.01)	21.39 (0.62)	20.16 (0.98)	21.84 (0.75)	28.65 (1.65)	
Mild	37.36 (0.01)	33.45 (0.79)	40.21 (1.28)	41.12 (0.93)	37.29 (1.78)	
Moderate	17.91 (0.01)	15.90 (0.54)	18.39 (0.93)	20.34 (0.70)	17.65 (1.59)	
Never	10.41 (0.01)	12.61 (0.46)	10.18 (0.79)	8.06 (0.68)	7.32 (0.96)	
Smoking (%)						< 0.0001
Former	24.94 (0.01)	24.94 (0.59)	26.11 (1.18)	24.67 (0.79)	22.88 (1.68)	
Never	55.56 (0.01)	50.23 (0.76)	57.45 (1.29)	62.14 (0.92)	52.19 (1.92)	
Now	19.49 (0.01)	24.83 (0.67)	16.44 (0.85)	13.19 (0.62)	24.93 (1.53)	
Diabetes (%)						< 0.0001
DM	14.34 (0.00)	19.68 (0.50)	13.11 (0.78)	8.81 (0.47)	7.83 (1.07)	
IFG	5.04 (0.00)	5.47 (0.31)	5.54 (0.57)	4.23 (0.33)	5.15 (1.02)	
IGT	3.45 (0.00)	4.28 (0.25)	3.21 (0.35)	2.63 (0.23)	2.32 (0.59)	
No	77.17 (0.02)	70.58 (0.60)	78.13 (1.04)	84.33 (0.60)	84.71 (1.77)	
CVD (%)						< 0.0001
No	96.60 (0.02)	95.60 (0.28)	97.20 (0.32)	97.50 (0.25)	97.63 (0.57)	
Yes	3.40 (0.00)	4.40 (0.28)	2.80 (0.32)	2.50 (0.25)	2.37 (0.57)	
Hypertension (%)						< 0.0001
No	62.94 (0.02)	55.08 (0.60)	63.56 (1.13)	71.61 (0.75)	72.65 (1.90)	
Yes	37.06 (0.01)	44.92 (0.60)	36.44 (1.13)	28.39 (0.75)	27.35 (1.90)	

PA, physical pattern; WW, weekend warrior; RA, regularly active; PIR, poverty income ratio; BMI, body mass index; BRI, body roundness index; CVD, cardiovascular disease.

### 3.2 Relationship between the PA pattern and AIP

The relationships between PA patterns and the AIP were analyzed via both univariate and multivariate linear regression, with results presented in **Table 2**. The analysis revealed that, compared with inactive adults, those engaged in regular physical activity exhibited a significant reduction in AIP levels across multiple model levels. Specifically, in all models analyzed, a statistically significant decrease in AIP levels was observed among regularly active adults, with β values of -0.11 (crude model, *P*<0.0001), -0.09 (partially adjusted model, *P*<0.0001), and -0.04 (fully adjusted model, *P*<0.0001). For insufficiently active adults, AIP reduction was significant only in the unadjusted model (β=-0.03, *P*<0.001) and was not significant in the adjusted models. Additionally, The WW activity pattern was associated with a minor reduction in AIP in the partially adjusted model (β=-0.03, *P*=0.02) but did not demonstrate consistent significance across all models. Importantly, when RA adults were used as the reference group, inactive, insufficiently active, and WW patterns were all significantly associated with increased AIP.

**Table 2:** Examination of the associations between PA patterns and AIP levels across all study participants.

Character	Crude model		Model 1		Model 2	
PA pattern	95%CI	*P*	95%CI	*P*	95%CI	*P*
Inactive	ref		ref		ref	
Insufficiently Active	-0.03 (-0.05, -0.01)	<0.001	-0.01 (-0.03, 0.00)	0.11	0.01 (-0.01, 0.03)	0.25
RA	-0.11 (-0.13, -0.10)	<0.0001	-0.09 (-0.11, -0.08)	<0.0001	-0.04 (-0.05, -0.03)	<0.0001
WW	-0.01 (-0.04, 0.02)	0.69	-0.03 (-0.06, 0.00)	0.02	0.01 (-0.02, 0.03)	0.59
*p* for trend		<0.0001		<0.0001		<0.0001
PA pattern						
RA	ref		ref		ref	
Inactive	0.11 (0.10, 0.13)	<0.0001	0.09 (0.08, 0.11)	<0.0001	0.04 (0.03, 0.05)	<0.0001
Insufficiently Active	0.08 (0.06, 0.10)	<0.0001	0.08 (0.06, 0.10)	<0.0001	0.05 (0.04, 0.07)	<0.0001
WW	0.11 (0.08, 0.14)	<0.0001	0.06 (0.04, 0.09)	<0.0001	0.05 (0.02, 0.07)	<0.0001
*p* for trend		<0.0001		<0.0001		<0.0001

PA, physical pattern; WW, weekend warrior; RA, regularly active; AIP, atherogenic index of plasma; CI, confidence interval;

Crude model

model 1: age, sex, race, marital status, PIR, education.

model 2: age, sex, race, marital status, PIR, education, BMI, BRI, drinking, smoking, Diabetes, CVD, Hypertension.

Notably, when RA adults served as the reference group, the inactive, insufficiently active, and WW patterns were all significantly linked to elevated AIP levels.

This finding emphasizes that the WW activity pattern did not provide the expected benefits in reducing AIP and may instead increase AIP risk due to a lack of daily activity. Thus, balanced and consistent daily physical activity is crucial for maintaining healthy AIP levels.

### 3.3 Subgroup analysis

Stratified analyses were conducted to examine the associations between physical activity levels and outcomes across different demographic characteristics (**Table 3**). The RA group consistently showed negative associations compared to the inactive group, with the strongest effects observed among participants aged 45-65 years (β=-0.05, 95%CI: -0.07 to -0.02, *P*<0.01) and males (β=-0.05, 95%CI: -0.07 to -0.03, *P*<0.01). The insufficiently active and WW groups showed minimal and largely non-significant differences compared to the inactive group across most strata. Tests for interaction revealed marginally significant effect modifications by education level (*P* for interaction=0.07) and marital status (*P* for interaction=0.09). Notably, the association between regular physical activity and outcomes was more pronounced among participants with more than high school education (β=-0.05, 95%CI: -0.06 to -0.04, *P*<0.0001) compared to those with less education. No significant effect modifications were observed by age (*P*=0.26), race (*P*=0.18), ethnicity (*P*=0.36), or income level (*P*=0.37), suggesting that the associations between physical activity levels and outcomes were generally consistent across these demographic characteristics.

**Table 3:** Relationships between PA patterns and AIP in different subgroups.

Character	Inactive	Insufficiently Active	*P*	RA	*P*	WW	*P*	*P* interaction
Age								0.26
≤45	ref	0.01 (-0.01, 0.03)	0.48	-0.04 (-0.05, -0.02)	<0.01	0.01 (-0.02, 0.04)	0.50	
45-65	ref	0.02 (-0.01, 0.05)	0.18	-0.05 (-0.07, -0.02)	<0.01	0.02 (-0.03, 0.07)	0.41	
≥65	ref	-0.02 (-0.05, 0.01)	0.14	-0.03(-0.06, 0.00)	0.03	-0.04 (-0.12, 0.03)	0.22	
Sex								0.18
Female	ref	0.01 (-0.01, 0.03)	0.53	-0.03 (-0.05, -0.02)	<0.01	-0.02 (-0.05, 0.02)	0.36	
Male	ref	0.02 (-0.01, 0.04)	0.22	-0.05 (-0.07, -0.03)	<0.01	0.01 (-0.02, 0.04)	0.51	
Race								0.36
Other	ref	0.01 (-0.03, 0.06)	0.50	-0.03 (-0.07, 0.00)	0.07	-0.01 (-0.10, 0.08)	0.88	
Non-Hispanic White	ref	0.02 (-0.01, 0.04)	0.16	-0.04 (-0.06, -0.02)	<0.01	0.01 (-0.02, 0.05)	0.39	
Mexican American	ref	-0.03 (-0.06, 0.00)	0.05	-0.02 (-0.05, 0.01)	0.25	0(-0.08, 0.08)	0.96	
Non-Hispanic Black	ref	-0.01 (-0.04, 0.01)	0.32	-0.04 (-0.07, -0.02)	0.002	-0.01 (-0.06, 0.03)	0.57	
Other Hispanic	ref	0.01 (-0.02, 0.05)	0.51	-0.05 (-0.08, -0.01)	0.01	0.01 (-0.05, 0.08)	0.74	
Marital status								0.09
Married or living with partner	ref	0.02 (0.00, 0.04)	0.07	-0.04 (-0.06, -0.03)	<0.01	0.02 (-0.01, 0.05)	0.24	
Never married	ref	0 (-0.04, 0.03)	0.93	-0.03 (-0.05, 0.00)	0.03	0 (-0.05, 0.05)	0.99	
Divorced, separated, or widowed	ref	-0.01 (-0.04, 0.02)	0.57	-0.03 (-0.06, 0.00)	0.03	-0.02 (-0.07, 0.04)	0.56	
Education								0.07
Completed high school	ref	0.02 (-0.02, 0.05)	0.34	-0.03 (-0.06, 0.00)	0.04	-0.01 (-0.06, 0.04)	0.66	
Less than high school	ref	0.02 (-0.01, 0.06)	0.18	0.01 (-0.02, 0.03)	0.71	0.02 (-0.03, 0.07)	0.48	
More than high school	ref	0 (-0.02, 0.02)	0.84	-0.05 (-0.06, -0.04)	<0.01	0.01 (-0.02, 0.04)	0.56	
PIR								0.37
Middle income	ref	0.02 (0.00, 0.05)	0.10	-0.03 (-0.05, -0.01)	0.003	0.02 (-0.01, 0.06)	0.20	
High income	ref	0 (-0.03, 0.03)	0.99	-0.05 (-0.07, -0.03)	<0.01	-0.02 (-0.06, 0.03)	0.44	
Low income	ref	0 (-0.02, 0.03)	0.79	-0.02 (-0.04, 0.00)	0.02	0.03 (-0.02, 0.09)	0.23	

PA, physical pattern; WW, weekend warrior; RA, regularly active; AIP, atherogenic index of plasma; CVD, cardiovascular disease; PIR, poverty income ratio.

Adjusted for age, sex, race, marital status, PIR, education, BMI, BRI, drinking, smoking, diabetes, CVD, hypertension.

### 3.4 Nonlinear relationship between total PA and AIP

**Figure 2:**
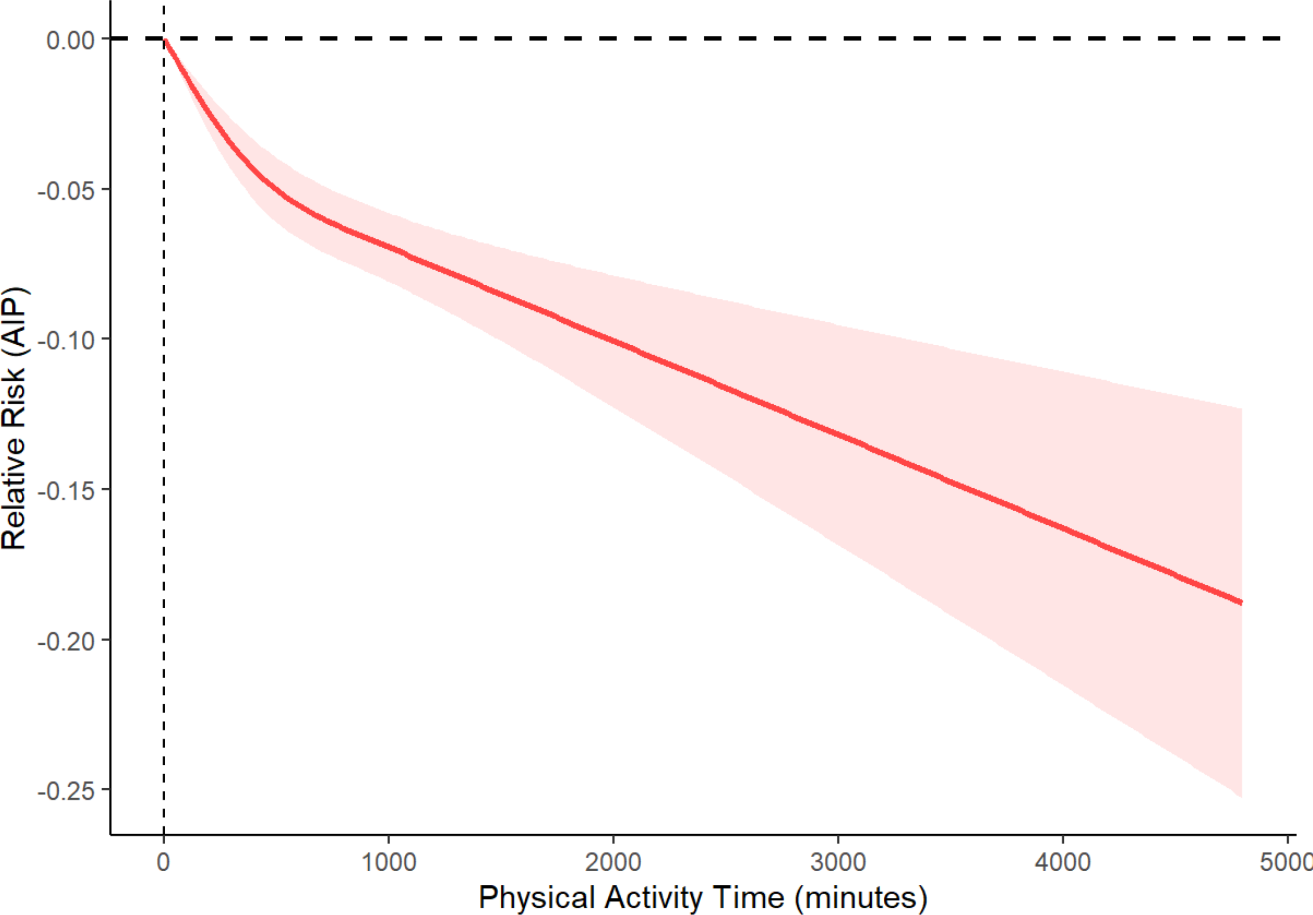
RCS analysis of the relationship between weekly aggregate PA and AIP across the entire participant cohort. The variables of age, sex, race, marital status, education, PIR, BMI, BRI, drinking, smoking, diabetes, CVD, hypertension were adjusted for during RCS analyses. Abbreviations: RCS, restricted cubic spline; PA, physical pattern; AIP, atherogenic index of plasma; CVD, cardiovascular disease; PIR, ratio of family income to poverty; BMI, body mass index.

Using the RCS analysis method, associations between the total amount of PA accumulated over a week and the AIP were investigated, as shown in **Figure 2**. The analysis indicated that as the total weekly PA duration increased, AIP levels exhibited a significant decreasing trend (*P* < 0.001, nonlinearity *P* < 0.001). A threshold effect analysis identified a critical point at 510 minutes of weekly total PA. Specifically, when the weekly total PA was below 510 minutes, each additional minute of PA was associated with a significant decrease in AIP (**Table 4**). However, when the weekly total PA exceeded the 510-minute threshold, the negative correlation between PA and AIP, while still statistically significant, showed a reduced rate of decrease in AIP.

**Table 4:** Threshold effect analysis of physical activity on AIP.

	β (95% CI)	*P*
One-line linear regression model	-0.000038 (-0.000049, -0.000028)	<0.001
Two-piecewise linear regression mode		
Inflection point (K)	510	
PA <K	-0.000081 (-0.00011, -0.000048)	<0.001
PA≥K	-0.000016 (-0.000033, 0.0000024)	<0.001
Log-likelihood ratio		0.00124

CI, confidence interval; PA, physical activity; AIP, atherogenic index of plasma. Outcome variable: AIP. Exposure variable: total PA. Adjusted for age, sex, race, marital status, PIR, education, BMI, BRI, drinking, smoking, diabetes, CVD, hypertension.

## 4 Discussion

Extensive research has confirmed the multifaceted positive effects of the WW phenomenon on individual health. Benefits include a significant reduction in the incidence of cardiovascular events, a lower risk of neurodegenerative diseases, positive effects on cognitive function, and an effective reduction in visceral fat accumulation, leading to improvements in abdominal and overall obesity (18, 25–28). A comprehensive meta-analysis further solidified this understanding, demonstrating that both the WW model and the RA model, compared with long-term inactivity, independently and significantly reduce the risk of cardiovascular diseases and all-cause mortality, with nearly equivalent effects (29).

However, this study revealed an intriguing and noteworthy deviation: although the RA group demonstrated a significant reduction in AIP, the WW group did not replicate this outcome, contrasting sharply with trends commonly reported in previous research. This phenomenon warrants further exploration. A primary factor may be attributed to differences in physical activity patterns. The RA group engages in regular and balanced physical activity, which plays a crucial role in maintaining low AIP levels. In contrast, the WW group participates in physical activity primarily on weekends, which lacks daily consistency. This intermittent activity pattern appears to have a more limited effect on metabolic health, failing to produce sustained and profound impacts on metabolic markers (30). Research has identified an exercise threshold beyond which significant metabolic responses are triggered, serving as an important defense against obesity and cardiovascular diseases. Studies indicate that engaging in 3 to 5 sessions of MPA per week, expending a total of 1200 to 1600 kcal, or the equivalent of 7 to 14 miles of exercise, significantly enhances beneficial metabolic changes in HDL-C, thereby reducing the risk of CHD-related mortality (31). Although the WW group may have met the recommended 150 minutes of weekly physical activity, this level might not have reached the critical threshold necessary for significant improvements in lipid metabolism.

Furthermore, a 24-week combined endurance and resistance training intervention study underscores the significance of regular physical activity. This study demonstrated improvements in weight and body composition among healthy women, as well as enhancements in carbohydrate and lipid metabolism, illustrating the essential role of regular exercise in mitigating the risk of metabolic diseases (32). Concurrently, several studies have highlighted the extensive benefits of regular moderate-intensity activities in optimizing lipid metabolism and reducing inflammatory responses (30, 33–35). Notably, moderate-intensity aerobic and resistance exercises, when performed at specific intensities and durations, can effectively improve liver fat degeneration. Although resistance training generally results in lower energy expenditure, its therapeutic effects are still substantial (36).

In summary, findings suggest that relying solely on short-term or intermittent exercise patterns (such as WWs) may be insufficient for achieving significant improvements in metabolic health. To effectively reduce AIP and prevent cardiovascular disease, a more regular and sustained physical activity pattern is crucial. Therefore, when designing personal exercise plans, it is important not only to consider the total duration of activity but also to emphasize the regularity, continuity, and appropriate intensity of the exercise. This approach is expected to maximize health benefits and ensure comprehensive metabolic optimization.

Of interest were the marginally significant effect modifications by education level and marital status. The more pronounced benefits observed among those with higher education levels might be attributed to several factors: better access to exercise facilities, greater health literacy, or more structured approaches to maintaining regular physical activity schedules (37, 38). Marital status also moderates the relationship between PA patterns and AIP. Due to shared living environments, similar behavioral patterns, and transferable lifestyle habits, married couples and cohabiting partners are more likely to develop stable physical activity habits (39, 40). Mutual encouragement and support between partners provide a strong social impetus for maintaining regular physical activity, which is crucial for significant improvements in AIP. The absence of significant effect modifications by age, ethnicity, and income level is equally informative, suggesting that the benefits of regular physical activity are relatively consistent across these demographic characteristics.

According to the 2020 physical activity guidelines published by the World Health Organization (WHO), adults should engage in at least 150 to 300 minutes of moderate physical activity (MPA), 75 to 150 minutes of vigorous physical activity (VPA) per week, or an equivalent combination of both to optimize overall health (13). This study’s threshold effect analysis identified 510 minutes of moderate-to-vigorous physical activity (MVPA) per week as a critical threshold for a significant reduction in AIP. Beyond this threshold, the improvement in AIP tends to plateau, indicating a diminishing marginal benefit of additional physical activity. Although further increases in activity still confer positive effects, their impact on significant further reductions in AIP is less pronounced. Therefore, aiming for at least 510 minutes of MVPA per week is recommended to achieve optimal reductions in AIP and effectively enhance cardiovascular health.

This study has notable strengths. Utilizing a large, nationally representative NHANES sample enhances the generalizability of the findings within the United States and provides a solid foundation for public health recommendations. The systematic analysis of diverse physical activity patterns offers a detailed understanding of their impact on AIP, adding valuable insights into cardiovascular health. Nevertheless, there are limitations. Reliance on self-reported physical activity data may introduce biases, such as recall or reporting bias. Future studies should incorporate objective monitoring tools, like wearable devices, to improve data accuracy. Additionally, the cross-sectional nature of this study precludes causal inferences. Longitudinal or randomized controlled trials are needed to better establish causality. Finally, the sample’s focus on American adults limits generalizability. Research in diverse international populations is necessary to evaluate the broader applicability of these findings.

## 5 Conclusion

This study offers a detailed examination of how different patterns of PA influence the AIP, a key marker of cardiovascular risk. The findings demonstrate that consistent, daily physical activity is more strongly associated with reduced AIP levels compared to intermittent, high-intensity activity concentrated on weekends. This highlights the significant role of sustained physical activity in mitigating cardiovascular risk factors.

These results carry substantial implications for both clinical practice and public health policy. The observed benefits of regular, moderate-intensity physical activity in lowering AIP suggest that healthcare providers should advocate for consistent exercise as part of routine care, with a particular emphasis on integrating daily PA into patient management plans. Given the strong association between daily activity and cardiovascular health, promoting regular, moderate-intensity exercise could be a more effective strategy for reducing cardiovascular risk than focusing on sporadic high-intensity bouts of activity.

From a public health perspective, the evidence calls for a shift in policy towards prioritizing daily physical activity in community-based interventions. Public health strategies should not only encourage physical activity but specifically promote its regular, daily engagement, as opposed to intermittent or weekend-only activity. Community programs, educational campaigns, and urban planning initiatives should be aligned to support daily PA through infrastructure improvements (e.g., pedestrian-friendly environments, accessible recreational facilities) and tailored interventions that encourage sustainable behavior changes.

By integrating these findings into public health frameworks, the potential to reduce cardiovascular disease burden on a population level is substantial. Such approaches could lead to long-term improvements in public health, with the added benefit of reducing healthcare costs associated with cardiovascular conditions.

## Data Availability

The original contributions presented in the study are included in the article/supplementary material. Further inquiries can be directed to the corresponding authors.
